# Impact of duration of treatments with metformin and sulfonylureas, individually or in combination, on diabetic retinopathy among newly diagnosed type 2 diabetic patients: a pooled cohort’s analysis

**DOI:** 10.1186/s40942-025-00637-w

**Published:** 2025-01-31

**Authors:** Mansour Bahardoust, Yadollah Mehrabi, Farzad Hadaegh, Davood Khalili, Ali Delpisheh

**Affiliations:** 1 Department of Epidemiology, School of Public Health & Safety, Shahid Beheshti University of Medical Sciences, Velenjak, 7th Floor, Bldg No.2 SBUMS, Arabi Ave, Tehran, Tehran Province Iran; 2 Prevention of Metabolic Disorders Research Center, Division of Biostatistics and Epidemiology, Research Institute for Endocrine Sciences, Shahid Beheshti University of Medical Sciences, Velenjak, Yaman St, Aarabi St, No.24, Tehran, Iran; 3 Safety Promotion and Injury Prevention Research Center, Shahid Beheshti University of Medical Sciences, Tehran, Iran; 4 Prevention of Metabolic Disorders Research Center, Research Institute for Endocrine Sciences, Shahid Beheshti University of Medical Sciences, Tehran, Iran

**Keywords:** Metformin, Sulfonylureas, Microvascular complications, Diabetic retinopathy, Type 2 diabetic patients

## Abstract

**Background:**

This study aimed to evaluate the effect of metformin and sulfonylurea (SUs) medication time on Diabetic retinopathy (DR) among newly diagnosed patients with type 2 diabetes (T2DM) using a pooled analysis. This study aimed to evaluate the effect of metformin and SUs’ medication time on DR among newly diagnosed T2DM using a pooled analysis.

**Methods:**

The data of 4,068 newly diagnosed DM individuals(mean age, 60.2 ± 0.85 years) from three prospective cohorts of Tehran Lipid and Glucose Study (TLGS), Multi-Ethnic Study of Atherosclerosis (MESA), and Atherosclerosis Risk in Communities (ARIC) with a mean age of 59.6 ± 08 years were pooled. The cumulative exposure to metformin, SUs, aspirin, statin, and anti-hypertensive medication was also determined using the same approach. The Cox proportional hazards (CPH) model was used to calculate the hazard ratio (HR) (95% CI) for the outcomes while adjusting for confounding factors such as fasting Blood Sugar (FBS), age, statin, aspirin, and anti-hypertensive medications.

**Results:**

During follow-up, DR occurred in 519 DM. Metformin alone, SUs alone, and the combination of both reduced the hazard of DR by 10%, 7%, and 11% for each year of use, respectively (*p* < 0.05). The protective effect of metformin and SUs, individually or in combination, on DR started approximately five years after the initial treatment and continued until approximately 15 years after the initial treatment and then reached a plato.

**Conclusion:**

Long-term treatment with metformin and SUs, individually and in combination, was associated with a reduced risk of DR in people with newly diagnosed diabetes for up to a decade compared with no treatment. These findings highlight the protective role of metformin and sulfonylureas as inexpensive and readily available drugs to prevent DR in people with newly diagnosed diabetes.

**Supplementary Information:**

The online version contains supplementary material available at 10.1186/s40942-025-00637-w.

## Introduction

Diabetes mellitus (DM), with 537 million adults between 20 and 79 years of age in 2021, is one of the most common endocrine diseases and is recognized as one of the major public health challenges worldwide [1–4]. According to recent studies, the incidence of diabetes is increasing, and it is predicted that this number will increase to 783 million people by 2045 [3, 5]. DM can lead to serious effects on the vital organs of the body and extensive outcomes, including the development of microvascular and Macrovascular complications [6].

Diabetic retinopathy (DR) is one of the most common microvascular complications related to DM, which is still one of the most common causes of blindness and vision loss in DM patients worldwide [7, 8]. With the increase in the prevalence of DM and the increase in years of life, the incidence of DR also increases significantly, which imposes a social and financial burden on any healthcare system [6, 7, 9–11]. More than 100 million people worldwide live with DR [12, 13]. DR occurs in approximately 30–40% of DM individuals [8, 13].

Many cases of vision loss caused by DR can be controlled with glycemic control [8, 14, 15]. Managing DM can decrease or postpone patient complications significantly [16]. High blood sugar and changes in metabolic pathways result in oxidative stress and the onset of nerve cell degeneration in the early phase of DR [17–19]. Damage to the blood vessels, the formation of small bulges in the blood vessels, and small areas of bleeding within the retina are initial indicators of non-proliferative diabetic retinopathy (NPDR). As the condition advances, the narrowing of blood vessels and blockages lead to twisted capillaries and reduced blood supply to the retina. In the late stage of DR, severe lack of oxygen leads to the growth of new blood vessels, bleeding into the vitreous, and detachment of the retina [17, 20, 21]. The growth of abnormal blood vessels in the retina can cause DR to be either proliferative (growing) or nonproliferative (not growing). Nonproliferative retinopathy, which is more common, may not necessitate treatment [22].

Metformin, the sole biguanide in oral antidiabetic (OAD) medications, is commonly utilized as the initial OAD in treating diabetic individuals due to extensive proof of its efficiency over the long term and its relatively lower occurrence of side effects, such as hypoglycemia, compared to the sulfonylurea (SUs) group [23]. Nevertheless, administering metformin alone to patients whose hemoglobin A1C (HbA1c) is < 7.5 could result in inadequate diabetes management. Thus, it should be combined with SUs drugs when prescribed to patients [24, 25]. Patients recently diagnosed with diabetes and whose condition is relatively steady are frequently managed by primary care physicians [26]. Therefore, for newly diagnosed DM patients, physician’s providers may prescribe oral antidiabetic drugs (OADs) like metformin and SUs based on various considerations [26]. These medications can benefit these patients by decreasing clinical indicators such as HbA1c levels or the risks of diabetes-related complications [27–29].

The efficacy of metformin and SUs has been demonstrated in various trials to decrease the microvascular complications and mortality linked to DM [30–33]. However, these trials are conducted under specific conditions and with specific population characteristics. Typically, the duration between diabetes onset and treatment initiation is uncertain in these populations, so the findings of these trials may not accurately reflect the drug’s effectiveness in the general population [34, 35]. In typical scenarios in local areas and the primary care facilities of each nation, these medications are frequently recommended for senior patients who are simultaneously dealing with multiple other illnesses for extended durations alongside other medications. The outcomes of taking these medications concurrently with other drugs in the community over the long term vary significantly from those observed in RCTs [34–39].

The effect of the duration of medication with metformin and SUs on DR by the duration of DM in long-term follow-up is still unclear. Therefore, considering the importance of this issue, this study, for the first time, evaluated the effect of medication time of metformin and SUs on DR by the duration of DM in newly diagnosed DM individuals with a pooled analysis of three prospective cohort studies.

## Methods

### Study design, setting, and population

The study, which accessed cohort data, was approved by the ethics committee of Shahid Beheshti University of Medical Sciences and the NHLBI Biologic Specimen and Data Repository Information Coordinating Center (BioLINCC). In this observational study, data from diabetic patients registered and followed up in the Tehran Lipid and Glucose Study (TLGS), Multi-Ethnic Study of Atherosclerosis (MESA), and Atherosclerosis Risk in Communities (ARIC) cohorts were extracted and combined.

**Fig. 1:**
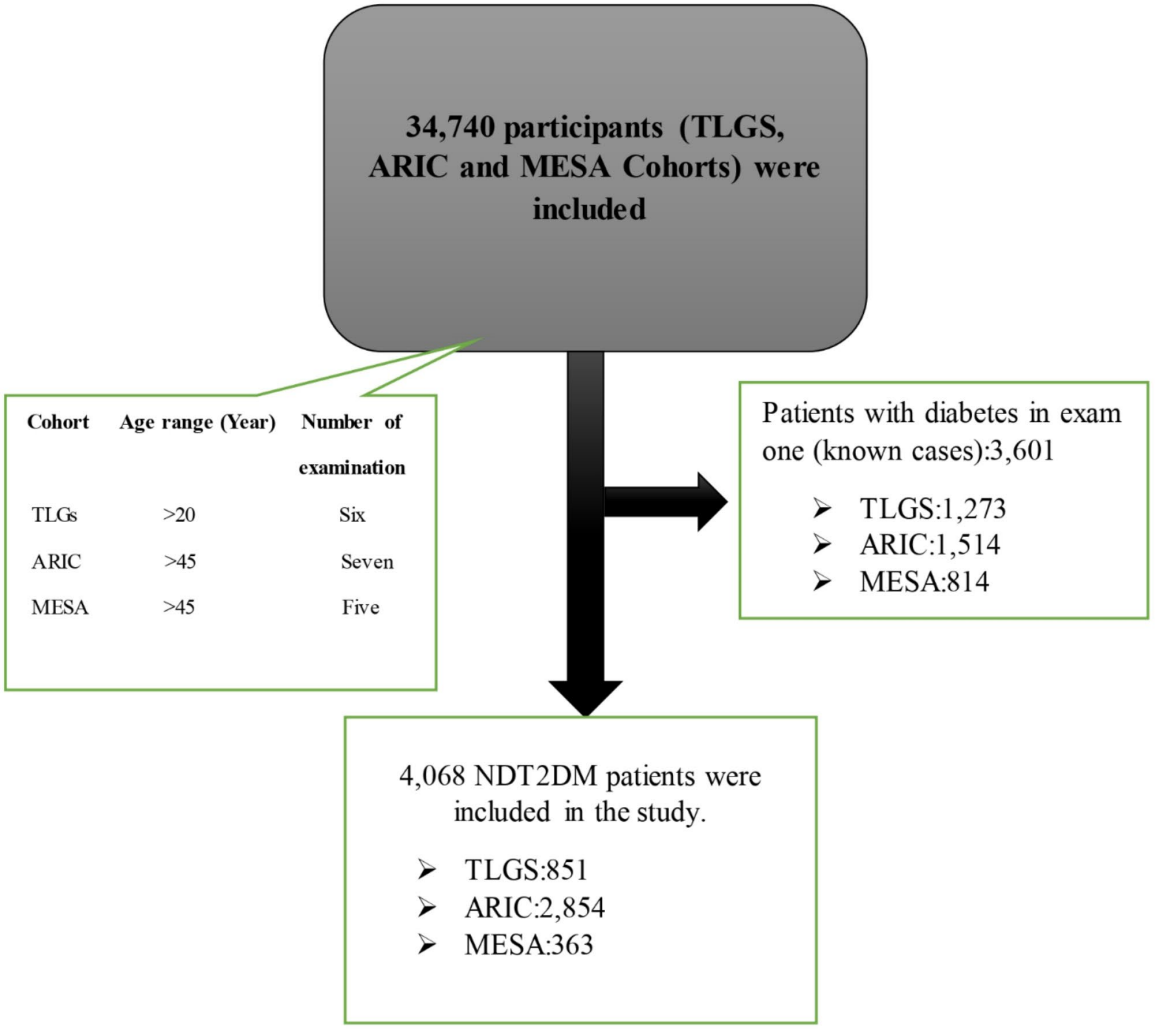
Flowchart of cohorts included in this study

During follow-up, patients with fasting blood sugar (FBS) levels greater than 126 mg/dL or those taking metformin, SUs, or a combination of both were defined as newly diagnosed DM individuals. Patients who had an FBS ≥ 126 at the first examination or were taking any antidiabetic medication were defined as known diabetes and excluded. Ultimately, 4,068 newly diagnosed DM individuals were included in the three cohorts during the follow-up period (Fig. 1).

### Inclusion and exclusion criteria

Inclusion criteria include patients with a definitive diagnosis of type 2 diabetes based on FBS ≥ 126, age ≥ 40 years, use of metformin or SUs (Glibenclamide or Gliclazide) or a combination of both, knowing the time of diabetes diagnosis, knowing the time of treatment initiation, knowing the time of medication time, knowing the time of occurrence of microvascular complications. Patients whose FBS levels were missing in each phase, concurrent use of other antidiabetic drugs (other OADs such as SGLT2 inhibitors or insulin), type 1 diabetes, suffering from any eye disorders before starting use OAD, diabetes known at the first examination and incomplete medical profile of patients were defined as exclusion criteria.

### Data management and extraction

Initially, a team of experts in epidemiology and endocrinology developed a three-part checklist for identifying the specific variables in the cohorts. The specific variables identified were determined based on the literature review and the study’s objectives. The definition and categorization of each variable were checked and validated across various cohorts. Because there were multiple definitions for each variable in each cohort, the same definition was used to extract each variable from all three cohorts. The most common definition between cohorts was used as the final definition to derive each variable.

The extracted variables in each cohort include four sections of demographic characteristics (age, sex, cohort start time, start time of each exam, end time of each exam, number of exams, sex, education level, smoking, body mass index (BMI), waist circumference (WC), marital status and physical activity (minutes per week)), clinical and laboratory findings (FBS, Triglyceride, cholesterol, low-density lipoprotein (LDL), high-density lipoprotein (HDL), systolic and diastolic blood pressure, creatinine, other medication time (aspirin, statin and anti-hypertensive), cumulative exposure (metformin, SUs and combination of both medication time (Year)) and outcome (DR).

### Diabetes duration calculation

The time to enter each exam in a given cohort and the interval between the two exams were calculated. Of course, the duration of each exam in the cohorts was calculated as an independent variable. The following steps were followed to calculate the desired times (the duration of diabetes until the outcomes occur and medication time). First, the duration of diabetes was calculated. Considering that all patients were healthy at the time of entry, if a person’s FBS was > 126 in the subsequent exam, the duration of diabetes was equal to 1/2 the duration of the previous exam until the outcome or the end of the cohort. For example, if a person’s FBS in exam 3 was > 126, the diabetes duration was equal to 1/2 the time of exam two until the occurrence of the outcome or the end of the cohort. Second, the time interval until the outcome occurs is calculated by subtracting the time of diabetes diagnosis from the occurrence of outcomes. Third, the time from the start of taking the medications to the time of changing or stopping the medications is defined as the medication time (cumulative exposure). For example, if a person was taking metformin in the current exam (For example, exam 2) but was taking SUs in the next exam, the metformin duration was equal to the total duration of exam 2 + 1/2 the duration of exam 3, with the assumption that the person will be half of the following exam has also taken metformin.

The method of calculating cumulative exposure and its underlying assumptions were consistent across all three cohorts and for all medications (metformin, SUs, combination of both, statin, anti-hypertensive, and aspirin).

Data for all time-dependent variables were collected for all exams, and the mean values from the time of diabetes diagnosis to the occurrence of the outcome were adjusted. This included the mean age, BMI, lipid profile (HDL, LDL, triglycerides, and cholesterol), FBS, waist circumference, physical activity, systolic and diastolic blood pressure, and creatinine up to the occurrence of any outcome or the last follow-up period. Cumulative exposure to statins, anti-hypertensive medications, and aspirin was also calculated.

### Exposure and outcome

The medication time, including metformin alone, SUs alone, and combinations of both, represented the exposure in patients during the cohorts. Since all individuals entering exam one were diabetes-free, the second exam of each cohort was considered the baseline for OAD consumption. The duration of each medication was calculated individually, and the interaction between metformin and SUs was estimated separately.

Diabetic retinopathy was the study outcome. Eye damage caused by T2DM (growth of abnormal blood vessels in the retina (proliferative and nonproliferative)) in one or both eyes was defined as DR based on an ophthalmologist’s diagnosis or laser eye surgery due to diabetes [22]. Diabetic retinopathy in the cohorts was performed based on fundus photography at each site by an ophthalmologist following a standard protocol. Screening periods in the cohorts were, on average, every 2 to 3 years.

### Statistical analysis

Stata statistical software version 17 was used for data analysis. Qualitative variables were described using % and frequency. Quantitative variables such as medication time (years), FBS, age, BMI, waist, lipid profile, blood pressure, disease duration, and creatinine level were presented with mean, quartiles, and standard deviation. The Cox proportional hazards (CPH) model was initially used to examine the linearity assumption in the relationship between exposure and outcomes using multivariable fractional polynomial (MFP) analysis. As a linear relationship between exposure and outcomes could not be established, the multivariable modeling with Cubic regression splines (MVRS) package was employed for the CPH analysis. Then, according to the method of P Royston et al. [40] With the fracplot command, the trend of changes in the hazard of occurrence of each outcome was plotted against the duration of medications. The number of knots was determined, and the slope of the outcome hazard changes with the medication time was estimated.

To assess the net effect of metformin and SUs, the separate impacts of each medication and the combined impact of both were computed using the Lincom package. The magnitude of the effect of exposure on each result was assessed in three different models. The first model was considered unadjusted (duration of metformin, SUs, and a combination of both). The second model included the crude model adjusted for the mean age and the mean FBS from the time of DM diagnosis to the occurrence of the outcome. The third model included model 2, adjusted for the variables of sex, smoke, waist, marital status, HDL, LDL, TG, education level, physical activity, cumulative exposure to other treatments (aspirin, statin, and anti-hypertensive), creatinine, and blood pressure. The impact of metformin and SUs medication timing on outcomes was reported using an adjusted hazard ratio (HR) with a 95% confidence interval (95% CI). A *p*-value of less than 0.05 indicated statistical significance.

## Results

### Demographic, laboratory, clinical characteristics, and mean medication time

The pooled mean age of patients at the time of DM diagnosis and during treatment in three cohorts (4,068 newly diagnosed DM) was 49.8 ± 1.3and 60.2 ± 0.85 years, respectively. 1969 (46.3%) of the patients were male. The mean FBS at the diagnosis was 159.6 ± 1.54 (range of 126.5 to 488). The mean physical activity of the patients during the follow-up was 75.2 ± 0.26 m/w. The history of aspirin and anti-hypertensive use was reported in 83.8% and 73.3% of patients, respectively. The mean medication time for metformin, SUs, and combination was 5.22 ± 0.41, 5.88 ± 0.6, and 4.88 ± 0.8, respectively. The mean FBS at the time of diagnosis and during the study was 160.6 ± 1.59and 148.6 ± 2.1 (mg/dl), respectively. (Table 1) The median follow-up was 15.84 ± 0.39 years.

**Table 1:** Demographic, laboratory, clinical characteristics, and duration of medication use in 4,253 newly diagnosed type 2 diabetes patients

Variables	Time	
	Diagnosis time	Mean in cohorts exams
**Democracy Characteristics** **(4,068 newly diagnosed DM)**		
Age (year)	49.8 ± 1.3 (40,79)	60.2 ± 0.85 (40,88)
Sex		
• Male • Female	1,883(46.3%) 2,185(53.7%)	-
Education level (Year)		
• Illiterate • < 8 • 8–16 • > 16	346(8.5%) 956(23.5%) 1,851(45.5%) 915(22.5%)	-
Smoke (yes)	1,997(49.1%)	-
Race		
• White • Black • Other	2,964 (69.7%) 846(19.9%) 443(10.4%)	-
Marital status(n)		
• Married • Unmarried • Divorced or widow	3,633 (89.3%) 199 (4.9%) 236(5.8%)	-
Aspirin use	3,551(83.8%)	-
Statin use	1,042 (24.5%)	-
Anti-Hypertensive Drugs	3,117(73.3%)	
BMI(kg/m^2^)	28.8 ± 0.32	27.9 ± 0.14
Waist(cm)	97.09 ± 0.23	101.2 ± 0.44
Physical activity(m/w)	69.4 ± 0.37	75.2 ± 0.26
**Clinical & Laboratory finding**		
FBS (mg/dl)	160.6 ± 1.59	148.6 ± 2.1
Cholesterol(mg/dl)	225.80 ± 1.3	198.4 ± 0.89
HDL(mg/dl)	58.3 ± 1.7	50.07 ± 0.45
Triglyceride(mg/dl)	177.5 ± 1.85	161.8 ± 1.7
Creatinine(mg/dl)	1.02 ± 0.007	1.06 ± 0.005
Systolic blood pressure (mmHg)	136.7 ± 1.4	131.2 ± 1.5
Diastolic blood pressure (mmHg)	82.2 ± 0.75	79.9 ± 0.63
**Mean Medication time in years (range)**		
Metformin	6.25 ± 0.41	(0.6, 14.2)
Sulfonylurea	5.86 ± 0.62	(0.7,11.4)
Sulfonylurea + Metformin	4.88 ± 0.8	(0.6,13.5)
Aspirin use	13.9 ± 2.3	(0.7,18.7)
Anti-Hypertensive Drugs	14.5 ± 21.99	(0.9,20.9)
Statin	10.11 ± 2.8	(0.6,17.9)

The mean FBS in patients who did not receive any antidiabetic medication during the study was significantly higher than the patients who received at least one antidiabetic medicine. No significant difference was observed between demographic and laboratory characteristics among the recipients of treatment regimens (Supplement 1-Table 1).

### Diabetic retinopathy

During follow-up, DR occurred in 519 newly diagnosed DM individuals. The mean time from diagnosis of DM to DR was 14.9 ± 0.18 years. Metformin and SUs, both alone and in combination in models 1 and 2, significantly reduced the risk of DR. In model 3, metformin alone (HR_Adj_: 0.90, 95% CI: 0.82, 0.98, P: 0.011), SUs alone (HR_Adj_: 0.93, 95% CI: 0.87, 0.99, P: 0.034) and their combination (HR_Adj_: 0.89, 95% CI: 0.80, 0.98, P: 0.001) significantly reduced the risk of DR. Also, the estimated effect of exposure to the outcome in the Fine-Gray model (competing risk) and the CPH model were almost similar (Table 2).

**Table 2:** The hazard ratio of the duration of using metformin and sulfonylurea on diabetic retinopathy

Model	Exposure (Medication time in years)	Cox model			Fine-Gray model		
		Hazard ratio	95% CI	*P* value	SHR	95% CI	*p*-value
**Diabetic Retinopathy**							
1	Metformin	0.87	0.81,0.94	0.001	0.90	0.84,0.97	0.001
	Sulfonylurea	0.90	0.82,0.99	0.022	0.93	0.84,1.02	0.08
	Sulfonylurea + Metformin	0.86	0.79,0.94	0.001	0.90	0.82,0.99	0.006
2	Metformin	0.88	0.80,0.96	0.001	0.92	0.86,0.98	0.012
	Sulfonylurea	0.91	0.85,0.98	0.025	0.94	0.88,1.02	0.12
	Sulfonylurea + Metformin	0.87	0.79,0.96	0.001	0.91	0.86,0.97	0.001
3	Metformin	0.90	0.82,0.98	0.011	0.93	0.87,0.99	0.015
	Sulfonylurea	0.93	0.87,0.99	0.034	0.94	0.87,1.00	0.038
	Sulfonylurea + Metformin	0.89	0.80,0.98	0.001	0.91	0.84,0.99	0.001

∗ Model 1: Crude

∗ Model 2: Adjusted for Mean of age and Mean of FBS at follow-up

∗ Model 3: Adjusted for Mean of age, FBS, BMI, statin, and smoking during the follow-up period

*The CPH model was used in models 1, 2, and 3

*Fine-Gray model was used to estimate the competing risk effect

**Fig. 2:**
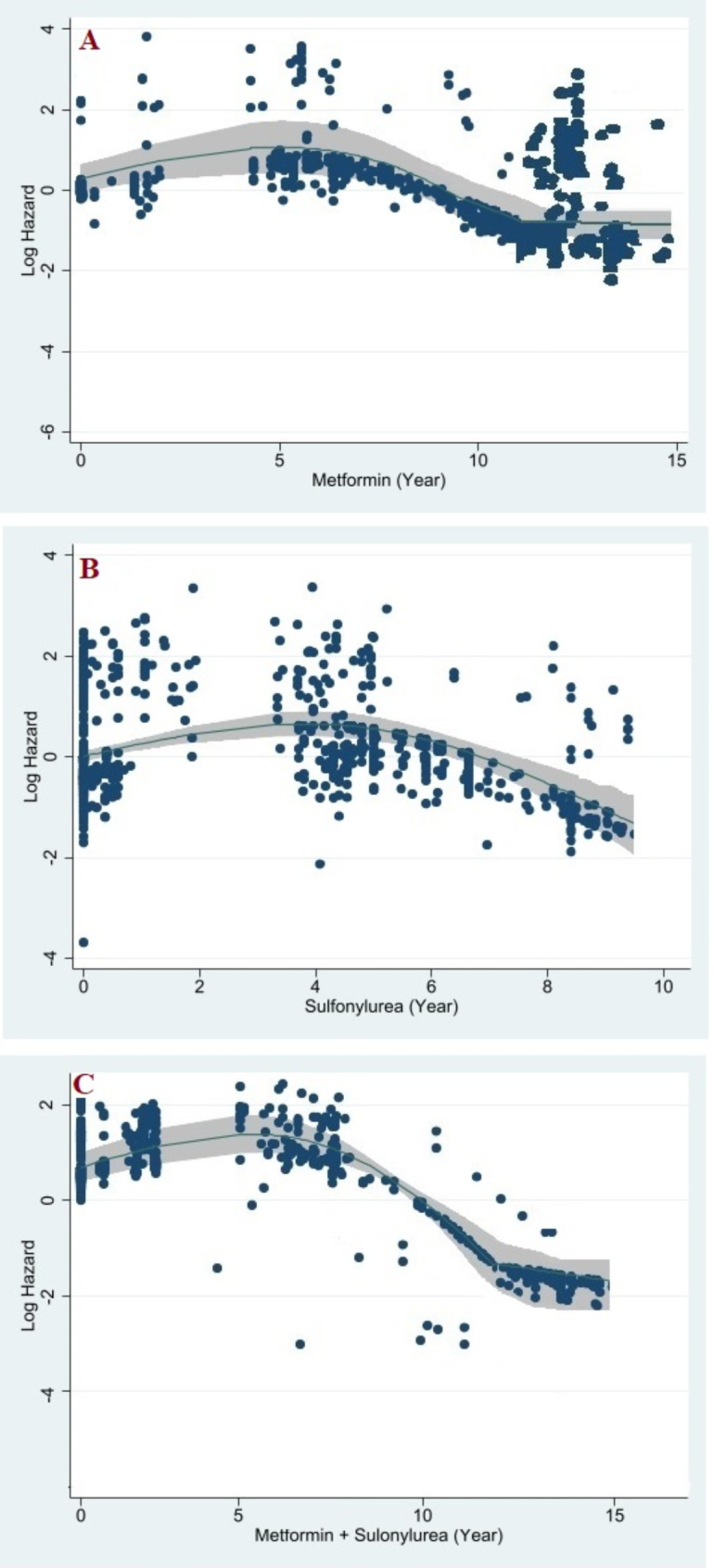
Fracplots show the hazard of DR for metformin (**A**), sulfonyl (**B**), and a combination of sulfonyl and metformin (**C**) over time

MVRS analysis adjusted for all variables showed that although the duration of use of all three treatment groups (individually and in combination) was significantly related to reducing the risk of DR, this relationship was not linear, and its reduction effect was different depending on the medication time. The protective effect of metformin on DR started almost 5 years after the initiation therapy, continued up to **12** years, and then remained plato (Fig. 2- Fracplot A). The protective effect of SUs medication on DR started approximately after 6 years and continued until 10 years after consumption, and then remained Plato (Fig. 2- Fracplot B). The protective effect of combination medication on DR started after 4 years and continued until 13 years after use. (Fig. 2 - Fracplot C)

Assuming a direct association between exposure and outcome and adjusting for all variables, metformin use resulted in a median 15% reduction in the risk of DR during follow-up (HRAdj: 0.85, 95% CI: 0.79, 0.93, P: 0.001). Using SUs reduced the risk of DR by an average of 13%. (HRAdj: 0.87, 95% CI: 0.80, 0.95, P: 0.001).

In addition, multivariate analysis showed that increasing mean age, FBS, BMI, and smoking were significantly related to increased DR hazard. At the same time, the use of Statins had a protective role (Data not shown).

## Discussion

In this study, using the data of 3 prospective cohorts, we evaluated the effect of metformin and SUs’ medication time on DR based on the duration of T2DM for the first time in 4,253 newly diagnosed DM individuals.

Our study showed that metformin and SUs, both individually and in combination, adjusted for other variables significantly reduced the risk of DR. The protective effect of metformin and SUs on DR started after a certain period of referees and continued for almost up to 10 years with different slopes. During follow-up, metformin and SUs reduced the risk of microvascular outcomes by an average of 15% and 13%, respectively. The protective effect of metformin and SUs alone and their combination on DR started approximately 5 years after initial treatment. Then, they continued with different gradients up to 15 years after initial treatment. The lack of effect of metformin and SUs (individually or in combination) on reducing the risk of DR in the first five years after the start of treatment in our study may be due to the low risk of this complication in the first years of DM. Because in this study, we evaluated newly diagnosed DM individuals, the risk of DR in these patients in the first years of the disease is intrinsically low, even without receiving medication. In the long term and with the increased risk of DR in DM, metformin and sulfonylureas had a protective effect on DR. The protective effect of metformin alone on DR started 5 years after the initiation of therapy, and its effect on reducing the risk of DR continued up to 12 years after use. The protective effect of SUs alone and their combination on reducing the risk of DR started almost 5 years after initiation therapy. The cohorts studied in this study were based on specific populations with specific characteristics, which were predominantly urban and had better access to health services than rural or deprived populations, and caution should be exercised in generalizing the results of this study to other populations. In addition to better access to health services, the patients studied in these studies may also differ in several other key variables that may be associated with the occurrence and progression of DR. Replication of these studies in other populations, especially deprived populations, may be associated with different results.

Several previous studies have reported the protective effect of metformin and SUs on diabetic retinopathy in DM [41–46]. However, according to our knowledge, the effect of these drugs based on the medication time in newly diagnosed DM individuals has not been reported. Therefore, these results still need to be discussed and are not definitive.

In line with the results of our study in 2022, Y Li et al., [42] showed that long-term use of metformin and SUs in DM significantly reduced the rate of severe nonproliferative or proliferative diabetic retinopathy (SNPDR/PDR). They showed that the odds ratio of SNPDR/PDR in patients who use metformin and SUs for a long time compared to diabetic patients who did not take antidiabetic drugs was 0.37 and 0.45, respectively. In another study, R Gabriel et al., [33] showed that glucose-lowering medications, including metformin, were significantly associated with reduced microvascular outcomes in people with prediabetes. The UK Prospective Diabetes Study [47] showed that sulfonylureas significantly reduced the risk of DR, in 3,867 newly diagnosed type 2 diabetes patients.

YP Fan et al., [43] showed that metformin significantly reduced the risk of NPDR in DM patients. They suggested that early administration of metformin can reduce the risk of DR in DM. They also showed that the protective effect of metformin in combination with other antidiabetics, including DPP-4i, was more significant in reducing the risk of NPDR. In our study, the protective effect of metformin against DR in combination with SUs was more significant than its individual effect. In a cohort study, AJ Barkmeier et al., [48] investigated the effectiveness of SUs on sight-threatening diabetic retinopathy (STDR) in 513,197 new DM patients. They showed that SUs were associated with a 39% reduction in the risk of STDR, which was consistent with the results of our study. In line with the results of our study, F Casanova et al., [49] showed that treatment with SUs and weight loss was significantly associated with a reduction in microvascular complications in DM patients.

In contrast, in a review of 19 RCTs, JG González-González et al., [30] showed that although metformin was associated with a decrease in the risk of DR compared to other anti-glucose-lowering drugs or placebo in DM, but this difference was not statistically significant and clinical evidence did not report the protective effect of metformin against DR. This incompatibility in results can be justified by the difference in the characteristics of the examined patients, sample size, follow-up period, and design of the two studies. In this review, they only examined the RCT studies that included DM with unknown disease duration and short follow-up period, while in our study, the long-term effects of metformin and SUs on microvascular complications based on the diabetes duration and medication time.

YR Chung et al., showed that metformin and SUs significantly reduced the risk of DR progression in individuals and combined with DPP4i in DM patients [50]. In this regard, JX Li et al., [45] showed that the simultaneous use of metformin and SGLT2 in the long term reduced the risk of DR progression, which confirmed the results of our study. Similar results were reported by J Hasselstrøm Jensen et al. [46]. In an umbrella review in 2024, L Tan et al., [51] showed that prescribing antidiabetic drugs on the risk of DR among people with type 2 diabetes is generally safe and may reduce the risk of DR. H Tang et al., [52] showed in a network meta-analysis, the current evidence shows that the relationship between inhibitors of SUs, DPP-4i, GLP-1RA or SGLT2 and the risk of DR in DM patients is unclear.

One of the mechanisms of reducing the risk of microvascular complications can be explained by controlling blood sugar and preventing hyperglycemia with metformin and SUs. KH Song et al., [53] showed that the reduction of mean glycaemia and dyslipidemia was significantly associated with reducing risk and preventing DR progression. Although the exact pathogenesis mechanism of DR is unclear, recent evidence suggests the prominent role of apoptosis, the involvement of vascular abnormalities, and cellular senescence in the pathogenesis of DR [17]. Additionally, disrupted autophagy contributes to the worsening of DR, highlighting the significance of autophagy in inhibiting the apoptosis or aging of retinal pigment epithelium (RPE) cells [17]. The increase in the AMP: ATP ratio under metabolic stress conditions like hypoxia and glucose deprivation triggers the AMP-activated protein kinase (AMPK) pathway, leading to the adjustment of cellular metabolism [54]. At a molecular level, metformin provides cytoprotection by activating the AMPK pathway [55]. This pathway regulates metabolism and shields cells from degradation and pathological changes linked to aging and DR. Recent findings have shown that metformin operates through both AMPK-mediated and non-AMPK-mediated pathways to produce effects extending beyond diabetes treatment, potentially preventing aging and improving conditions related to DR [56, 57]. Besides the AMPK pathway, recent findings indicate that metformin can control pathways not dependent on AMPK, including autophagy, oxidative stress, and ER stress. This helps in protecting retinal cells from vascular abnormalities, apoptosis, and cell senescence, ultimately preventing the development of DR [54, 58].

Given that the design of the three cohorts (TLGS, MESA, and ARIC) was similar in terms of the characteristics of the populations under study (new diabetic patients), the variables considered as confounders, follow-up intervals, exposure (duration of treatment with metformin and sulfonylurea), and outcome (DR), we pooled the data from these three cohorts to increase the accuracy of the study. Common definitions were used to measure the variables in all three cohorts. We also analyzed the results of all three cohorts separately, and their individual results were consistent, indicating the validity of the studies. However, there were limitations in conducting the study, as noted.

### Limitations

Our study had strengths and weaknesses that should be considered. First, we were unaware of the type of SU medication in each cohort, so we couldn’t discuss the safety issue according to the SU class. Also, due to the high missing in HbA1c, we could not estimate HbA1c, and the definition of DM was based on FBS. However, given that the aim was to evaluate the effect of treatment duration on the outcome and FBS was also measured and recorded during the follow-up period in all phases, its negative effect on the results may be very limited and clinically negligible. According to the definitions of the data recorded in the cohorts, we could not separate the results based on the type of retinopathy (nonproliferative or proliferative retinopathy). Unfortunately, medication adherence in cohort studies is self-reported. However, examining the trend of FBS in different phases can indicate the level of medication adherence. Continuous follow-up of patients in a combined manner (face-to-face and telephone follow-up), shortening follow-up periods and ease of performing blood sugar tests, and using more accurate tests (e-One) in shorter follow-up periods can provide more accurate medication adherence results.

The study’s main strength was evaluating the effect of metformin and SUs’ medication time on DN and DR in a large sample with a long follow-up period while adjusting for confounders in NDM patients.

## Conclusion

Among newly diagnosed diabetic patients, long-term treatment with metformin and SUs alone and in combination was associated with a reduced risk of DR for about a decade compared with no treatment (patients who did not receive any antidiabetic drug). The protective effect of metformin and SUs on DR started after a certain trial period and continued for almost 10 years with different gradients. The protective effect of metformin and SUs alone and their combination on DR started approximately 5 years after initial treatment and then continued with different slopes up to 15 years after initial treatment. The combination therapy of Metformin and SUs can still be used with reasonable effectiveness especially in the first years of diabetes diagnosis. These cohorts were mainly conducted on predominantly urban patients with specific characteristics, and caution should be exercised in generalizing the results of this study to other populations. Replication of these studies in other populations, especially disadvantaged populations, may be associated with different results.

## Electronic supplementary material

Below is the link to the electronic supplementary material.

Supplementary Material 1

## Data Availability

The datasets generated or analyzed during the current study are available from the corresponding author on reasonable request.
