# Correction: Impact of duration of treatments with metformin and sulfonylureas, individually or in combination, on diabetic retinopathy among newly diagnosed type 2 diabetic patients: a pooled cohort’s analysis

**DOI:** 10.1186/s40942-025-00658-5

**Published:** 2025-03-12

**Authors:** Mansour Bahardoust, Yadollah Mehrabi, Farzad Hadaegh, Davood Khalili, Ali Delpisheh

**Affiliations:** 1 Department of Epidemiology, School of Public Health & Safety, Shahid Beheshti University of Medical Sciences, Velenjak, 7th Floor, Bldg No.2 SBUMS, Arabi Ave, Tehran, Tehran Province Iran; 2 Prevention of Metabolic Disorders Research Center, Division of Biostatistics and Epidemiology, Research Institute for Endocrine Sciences, Shahid Beheshti University of Medical Sciences, Velenjak, Yaman St, Aarabi St, No.24, Tehran, Iran; 3 Safety Promotion and Injury Prevention Research Center, Shahid Beheshti University of Medical Sciences, Tehran, Iran; 4 Prevention of Metabolic Disorders Research Center, Research Institute for Endocrine Sciences, Shahid Beheshti University of Medical Sciences, Tehran, Iran

**Correction: Int J Retin Vitr 11, 9 (2025).**

10.1186/s40942-025-00637-w.

Following publication of the original article [1], the authors reported the following errors:

The affiliation of Davood Khalili is incorrect. The correct affiliation is: Prevention of Metabolic Disorders Research Center, Research Institute for Endocrine Sciences, Shahid Beheshti University of Medical Sciences, Tehran, Iran.

Further to this, it an error was reported in the Abstract, under the Methods heading. Tehran Sugar and Lipid Study (TLGS) should be Tehran Lipid and Glucose Study (TLGS).

The original article [1] has been corrected.
