# Supplementary material for: Impact of duration of treatments with metformin and sulfonylureas, individually or in combination, on diabetic retinopathy among newly diagnosed type 2 diabetic patients: a pooled cohort’s analysis
Source: Int J Retina Vitreous. 2025 Jan 31;11:9. doi: 10.1186/s40942-025-00637-w (PMC11784098; doi:10.1186/s40942-025-00637-w)
Supplement: Supplementary file 1 — Supplementary Material 1 [file 40942_2025_637_MOESM1_ESM.docx]

Table1: Comparison of demographic, clinical and laboratory characteristics based on drug groups

| Variables | Metformin  1,252 | Sulfonylurea  746 | Combination  1,463 | No  607 | P value |
| --- | --- | --- | --- | --- | --- |
| Age (year) | 57.6±9.6 | 60.22±8.1 | 60.1±7.6 | 60.9±5.9 | 0.42 |
| Sex   - Male - Female | 601(48/%)  651(52%) | 344(46.1%)  402(53.9%) | 670(45.8%)  793(54.2%) | 270(44.5%)  337(55.5%) | 0.35 |
| Education level (Year)   - <8 - 8-16 - >16 | 397(31.7%)  568(45.4%)  287(22.9%) | 211(28.3%)  349(46.8%)  86(24.9%) | 452(30.9%)  655(44.8%)  356(24.3%) | 240(39.5%)  279(46%)  88(14.4%) | 0.068 |
| Smoke (yes) | 646(51.6%) | 380(51%) | 696(47.6%) | 274(45.2%) | 0.071 |
| Marital status(Married) | 1,148(91.7%) | 681(91.2%) | 1,324(90.5%) | 480(79.1%) | 0.08 |
| BMI(kg/m2) | 29.36±4.5 | 29.2±4.2 | 28.9±4.4 | 27.99±4.47 | 0.13 |
| Waist(cm) | 101.2±9.1 | 100.4±9.9 | 101.8±8.3 | 102.6±7.31 | 0.18 |
| Physical activity(m/w) | 59.01±10.3 | 56.56±11.2 | 65.2±9.8 | 58.6±10.1 | 0.15 |
| Clinical & Laboratory finding | | | | |  |
| FBS (mg/dl) | 145.3±29.18 | 143.3±23.2 | 142.3±21.1 | 163.3±18.4 | 0.029 |
| Cholesterol(mg/dl) | 200.1±29.4 | 195.3±22.7 | 194.9±22.9 | 195.4±18.95 | 0.28 |
| LDL(mg/dl) | 119.24±22.4 | 123.2±20.5 | 118.4±25.1 | 126.35±19.5 | 0.43 |
| HDL(mg/dl) | 48.7±11.4 | 54.12±11.2 | 54.13±10.6 | 54.46±11.03 | 0.34 |
| Triglyceride(mg/dl) | 177.4±44.2 | 178.6±47.1 | 162.6±51.2 | 167.3±54.2 | 0.11 |
| Creatinine(mg/dl) | 1.054±0.15 | 1.06±0.19 | 1.04±0.22 | 1.07±0.16 | 0.62 |
| Systolic blood pressure (mmHg) | 111.74±13.3 | 129.4±18.3 | 128.1±18.9 | 136.02±19.2 | 0.12 |
| Diastolic blood pressure (mmHg) | 77.4±7.9 | 73.2±10.7 | 73.3±8.6 | 72.3±7.94 | 0.66 |
| Mean Medication time in years (range) | | | | |  |
| Aspirin | 13.8±4.6 | 13.6±4.1 | 14.1±4.1 | 10.4±3.2 | 0.032 |
| Statin | 10.3±3.5 | 12.3±2.8 | 12.8±3.9 | 8.11±4.2 | 0.011 |
| Anti-hypertensive | 14.3±3.1 | 14.4±3.8 | 14.05±3.9 | 11.34±4.6 | 0.027 |
| Outcome | | | | |  |
| Retinopathy( yes) | **146(11.7%)** | **85(11.4%)** | **159(10.9%)** | **129 (21.3%)** | 0.006 |

*Overlap over 6 months was defined as the dominant drug.
